# Proteome Changes in the Plasma of Myelodysplastic Syndrome Patients with Refractory Anemia with Excess Blasts Subtype 2

**DOI:** 10.1155/2014/178709

**Published:** 2014-05-25

**Authors:** Pavel Majek, Zuzana Riedelova-Reicheltova, Jiri Suttnar, Klara Pecankova, Jaroslav Cermak, Jan E. Dyr

**Affiliations:** Institute of Hematology and Blood Transfusion, U Nemocnice 1, 128 20 Prague 2, Czech Republic

## Abstract

The goal of this study was to explore the plasma proteome of myelodysplastic syndrome (MDS) patients with refractory anemia with excess blasts subtype 2 (RAEB-2) in comparison to healthy controls. 20 plasma samples were separated with 2D electrophoresis and statistically processed with Progenesis SameSpots software. 47 significantly differing (*P* < 0.05) spots were observed, and 27 different proteins were identified by nano-LC-MS/MS. Mass spectrometry-based relative label-free quantification showed a 2-fold increase of the leucine-rich alpha-2-glycoprotein (LRAG) peptide levels in the RAEB-2 group. Changes in the fragments of the inter-alpha-trypsin inhibitor heavy chain H4 (ITIH4) protein were observed. Western blot analysis showed no differences in albumin and ITIH4 levels, while increased expression was observed for LRAG in the RAEB-2 group. Quantification using ELISA showed decreased plasma level of alpha-2-HS glycoprotein in the RAEB-2 group. In conclusion, this is the first time that alpha-2-HS glycoprotein and LRAG were proposed as new biomarkers of RAEB-2 and advanced MDS, respectively. Alpha-2-HS glycoprotein, a protein involved in the bone marrow development and previously proposed as a MDS biomarker candidate, was significantly decreased in RAEB-2. Increased expression and changes in modification(s) were observed for LRAG, a protein involved in granulocytic and neutrophil differentiation, and angiogenesis.

## 1. Introduction

Refractory anemia with excess blasts subtype 2 (RAEB-2) belongs to the most severe subgroups of myelodysplastic syndrome (MDS), a group of heterogeneous oncohematological bone marrow disorders characterized by ineffective hematopoiesis, blood cytopenias, and a progression of the disease toward acute myeloid leukemia (AML). In particular the progression of MDS toward AML is observed in advanced MDS subgroups (as RAEB-2) and represents a serious feature correlating with poor patient outcome. The molecular mechanisms of MDS progression, together with the disease genesis and development, are not yet fully understood. Although there is some knowledge at the DNA level [1–4], MDS proteomics is still at an early stage. Proteomic studies may provide new insight into disease mechanisms impossible to see at the DNA level (protein modifications, complexes, etc.). Moreover, plasma proteomics can identify biomarker candidates useful in clinical practice, especially in combination with other analytical techniques like surface plasmon resonance capable of producing high-throughput biosensors [5–7]. Considerable effort has been expended in the preparation of such biosensors in recent years [8].

In three previous proteomic studies of different MDS subgroups, plasma proteome changes were interrogated in MDS patients with refractory anemia, refractory anemia with ringed sideroblasts (RA-RARS) [9], refractory cytopenia with multilineage dysplasia (RCMD) [10], and refractory anemia with excess blasts subtype 1 (RAEB-1) [11]. Therefore, this study represents an important part to cover the full range of different MDS risk subgroups from very low to high, according to the WHO classification-based prognostic scoring system (WPSS) for MDS [12]. The goal of this study has been to explore the plasma proteome of MDS patients with RAEB-2, in comparison with that of healthy controls.

## 2. Materials and Methods

Blood samples were collected as described previously [10]. All tested individuals agreed to participate in the study on the basis of an informed consent. All samples were obtained and analyzed in accordance with the Ethical Committee regulations of the Institute of Hematology and Blood Transfusion. A total of 8 patient plasma samples and 12 healthy controls have been investigated in this proteomic study. The diagnosis of RAEB-2 was established according to the WHO classification criteria [13]. The age of the patients ranged from 49 to 79 years; the healthy control donor age ranged from 21 to 36 years. The patient and control groups included 3 males (38%) and 5 males (42%), respectively.

This study followed the methods used in our previous MDS studies [9–11]. 150 *μ*L of diluted plasma (1 : 3 in MARS depletion buffer) was used to deplete 14 high-abundant plasma proteins (MARS Hu-14 column; Agilent, Santa Clara, CA, USA). This corresponded to the 37.5 *μ*L volume of undiluted plasma used for each sample in this study. Proteins were separated by isoelectric focusing (immobilized pH gradient strips pI 4–7, 7.7 cm) and then by SDS-PAGE (8 × 10 cm, 10% resolving gel, 3.75% stacking gel, and 30 mA/gel). Gels were stained with colloidal Coomassie and scanned and processed with Progenesis SameSpots software (Nonlinear Dynamics, Newcastle upon Tyne, UK) that computed the fold and *P* values of all spots using one-way ANOVA analysis, and principal component analysis (PCA) was performed. PCA was performed using only the spots of statistical significance (based on 2D SDS-PAGE) employed for protein identification. No technical replicates were used for 2D SDS-PAGE (only nonpooled individual samples of patients and donors were used). MS/MS mass spectrometry (HCT ultra-ion-trap mass spectrometer with nanoelectrospray ionization; Bruker Daltonics, Bremen, Germany) coupled to a nano-LC system (UltiMate 3000; Dionex, Sunnyvale, CA, USA) was used to perform MS analysis. Mascot (Matrix Science, London, UK) was used for database searching (Swiss-Prot). Two unique peptides (with a higher Mascot score than the minimum for identification, *P* < 0.05) were necessary to successfully identify a protein. All the procedures above have been described in detail in our previously published literature [9–11, 14].

Relative label-free protein quantification was used to compare the tryptic peptide levels of leucine-rich alpha-2-glycoprotein (LRAG) in the patient and control groups. Two LRAG peptides were monitored: ENQLEVLEVSWLHGLK with precursor ion 947.5* m/z* (charge 2+) and product ion 1181.8* m/z* (y10); TLDLGENQLETLPPDLLR with precursor ion 1019.1* m/z* (charge 2+) and product ion 710.4* m/z* (y6). Extracted ion chromatograms of the product ions were generated with an ion* m/z* width of ±0.5 Da, and peak areas were calculated after automatic integration using DataAnalysis software (version 4.0; Bruker). Each sample was measured twice, and a *t*-test was used for statistical analysis. All sample results were validated according to MS/MS spectra and retention times [15]. The procedure utilized the acetonitrile precipitation of plasma proteins and trypsin digestion protocol by Kay et al. [16] and was described in detail previously [9, 11].

Western blot analysis was performed as previously described [17]. Proteins were transferred from a gel to a polyvinylidene fluoride (PVDF) membrane (10 V constant voltage for 1 hr); the membrane was then incubated with a blocking buffer (3% bovine serum albumin in phosphate buffered saline) at 4°C overnight, rinsed, and incubated with a primary antibody at 30°C for 45 min (monoclonal mouse anti-leucine-rich alpha-2-glycoprotein, 1 : 400, Abcam, Cambridge, UK; monoclonal mouse anti-albumin, 1 : 2000, Sigma-Aldrich, Prague, Czech Republic; monoclonal mouse anti-ITIH4, 1 : 2000, Abnova, Taipei, Taiwan). After rinsing, the membrane was incubated with a secondary antibody at 30°C for 45 min (rabbit anti-mouse IgG antibody conjugated with peroxidase, 1 : 60000, Sigma-Aldrich). After rinsing, a chemiluminescent substrate (SuperSignal West Pico; Thermo Scientific, Waltham, MA, USA) was added to the membrane for 5 min, and an appropriate film exposure (Amersham Hyperfilm ECL; GE Life Sciences, Piscataway, NJ, USA) was performed [9–11]. Pooled patient (all 8 RAEB-2) and control (8 sex-matched) samples were used for western blotting analysis. Analyzes were compared using ImageJ software (http://imagej.nih.gov/ij/) [18].

Alpha-2-HS glycoprotein (A2HSG) plasma levels were measured using a commercial ELISA kit (Abcam) [9]. Sex-matched samples were measured according to manufacturer instructions; results were expressed as means ± standard deviations. An unpaired *t*-test was used for comparison of the plasma level between the RAEB-2 patient and control groups [9].

## 3. Results

**Figure 1:**
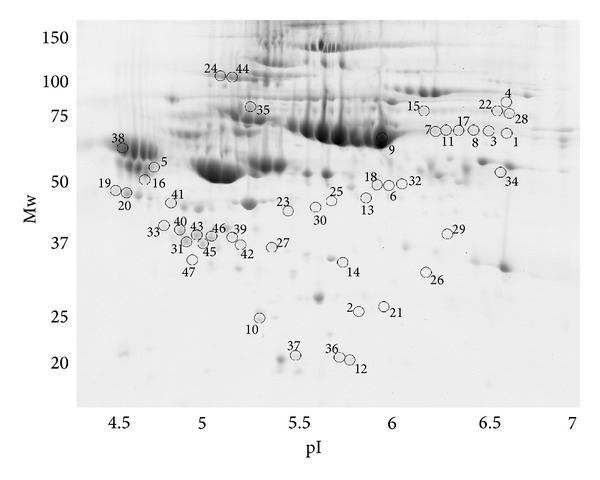
Positions of all the 47 unique spots that differed significantly (*P* < 0.05) in their normalized volumes when the RAEB-2 and control groups were compared are displayed on an illustrative 2D gel of a patient sample.

Comparing the patient RAEB-2 group (*n* = 8) with the healthy control group (*n* = 12) using two-dimensional electrophoresis (2-DE) gels, we found 47 unique spots that differed significantly (*P* < 0.05) in their normalized volumes (Figure 1). Proteins in 43 spots were identified; they corresponded to 27 different proteins. The list of all spots, including ANOVA *P* values, their multiplication (fold value), protein identification with the number of identified peptides (unique peptides above the identity threshold score), protein accession number (Swiss-Prot), and the sequence coverage, is summarized in Table 1.

**Figure 2:**
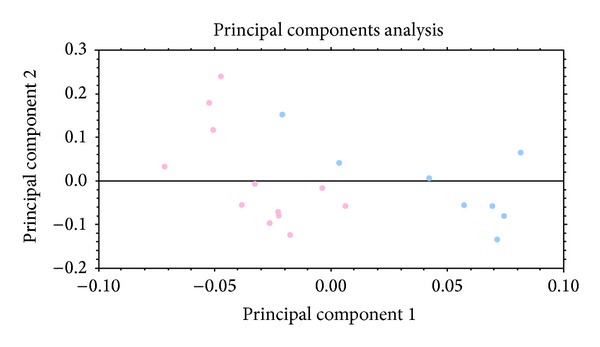
Principal component analysis based on spots that significantly differed (*P* < 0.05) between the compared groups was performed to assess whether the grouping of patient and healthy controls (based on 2D SDS-PAGE) reflects their stratification using classical clinical diagnosis [9–11]. PCA showed that the samples separated into two sample groups corresponding to the RAEB-2 (blue dots) and healthy control groups (pink dots).

Principal component analysis (PCA) was performed making use of Progenesis SameSpots software, employing only the spots of statistical significance (based on 2D electrophoresis), in order to assess whether grouping of patients and healthy controls based on the 2D SDS-PAGE results reflects their stratification using clinical diagnosis [9–11]. PCA showed clear separation of the samples (2-DE gels) into two groups corresponding to the patient (blue dots) and the control (pink dots) groups, respectively (Figure 2).

Leucine-rich alpha-2-glycoprotein (LRAG) was selected for relative label-free protein quantification, as there was no coidentification within its spot (spot 20), and the normalized spot volume changed more than 50%. As it is possibly related to MDS and its pathophysiology and the same LRAG peptides were quantified in the previous study of the RAEB-1 subgroup [11], the results were compared. All the RAEB-2 patient samples (*n* = 8) and control samples (*n* = 12) were used for relative quantification. There was an approximately twofold increase in both peptide levels in the RAEB-2 subgroup, when compared to the control group: 1.92-fold increase for ENQLEVLEVSWLHGLK (947.5/1181.8) and 2.01-fold increase for TLDLGENQLETLPPDLLR (1019.1/710.4). The differences were not found to be statistically significant.

**Figure 3:**
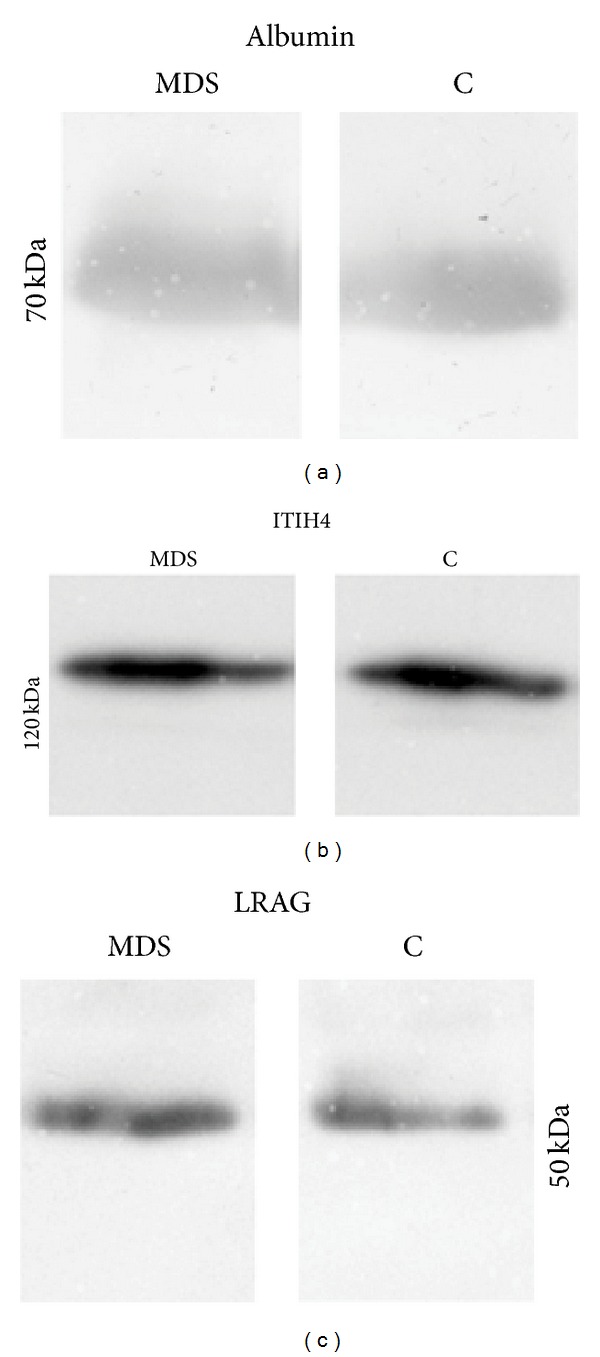
Western blot analysis was performed for three different proteins (1D SDS-PAGE, 12% resolving gel): albumin (a), ITIH4 (b), and LRAG (c). No difference was observed for albumin and ITIH4, while an increase (approximately 30%) was observed for LRAG in the RAEB-2 group as estimated using ImageJ software. Pooled samples (pooled equal volumes of individual samples) were used for patient (MDS) and healthy control (C) groups.

Western blot analysis of inter-alpha-trypsin inhibitor heavy chain H4 (ITIH4) showed no differences in the intact protein level between the RAEB-2 and control groups; ITIH4 fragments were not detected. No differences were observed in the plasma level of human serum albumin. Western blotting showed an increase in the LRAG plasma level in the RAEB-2 patient group—the increase corresponded to approximately 30% when compared with the control group. Western blotting results are illustrated in Figure 3.

The A2HSG plasma level was measured for all patient RAEB-2 samples (*n* = 8) and the same number of sex-matched control samples (*n* = 8). The A2HSG level was estimated to be 438 ± 55 *μ*g/mL for RAEB-2 samples and 545 ± 48 *μ*g/mL for controls. The difference was found to be statistically significant (*t*-test, *P* = 0.00103).

## 4. Discussion

Myelodysplastic syndrome is a group of heterogeneous oncohematological bone marrow disorders. This heterogeneity is related not only to genetic, proteomic, or morphological changes but it is manifested in outlook, life quality, or survival of patients. For example, the low-risk MDS subgroups are characterized by good survival (years), while typical survival for severe MDS subgroups (like RAEB-2) counts in months. Moreover, MDS can rapidly progress toward more severe subgroups or AML. Therefore, it is of the utmost importance to search for new biomarkers that could be used for MDS diagnostics and the monitoring of the disease progression.

This study describes plasma proteome changes in the high-risk RAEB-2 subgroup of MDS and thus completes the picture of plasma protein alterations with regard to the previous MDS subgroup studies [9–11]. Contrary to previous studies of low- or intermediate-risk MDS subgroups, this study interrogates the high-risk subgroup RAEB-2 and thus has potential to find protein markers highly valuable for monitoring the disease development. Comparing the overall results with respect to the number of changes (spots) that were identified in all four studies, it is obvious that there is no correlation (numbers of spots) with WPSS score, as has been pointed out previously [9]. Again, it seems that “a depth of changes” is the principal factor; however, the limitations of the methods used have to be considered, especially the limited level of protein detection. Thus, the situation may dramatically change when low-abundance proteins are investigated. In this study, the same characteristics for the control group (young healthy donors) were used as in the previous studies [9–11] to maintain consistency in the results and their interpretation.

Fragmentation of two proteins—ITIH4 and albumin—was observed in this study. Changes in the spots containing ITIH4 fragments were observed in all of the previous MDS subgroup studies, in which ITIH4 properties were described in detail [9–11]. It is known that MDS is linked to higher proteasome activity in plasma [19]—this could be an explanation for the results observed as the spots with ITIH4 fragments (corresponding to those physiologically produced by plasma kallikrein cleavage) showed a decrease in their volumes across different MDS subgroups. The decrease could be caused by additional fragmentation producing small fragments undetectable by 2-DE as designed in this study. In accordance with the results obtained in previous studies, there was no difference observed in ITIH4 expression (Figure 3). In general, it seems that there are observable changes in ITIH4 fragments in different MDS subgroups independent of ITIH4 expression (which remained unchanged). The decrease of spot volume in spots containing albumin fragments could be also explained by higher proteasome activity in MDS [19]. Another factor could be acute phase reaction; however, no obvious difference was observed in western blot analysis (Figure 3). The albumin changes cannot be explained easily, though a high-abundant plasma protein depletion step during the sample preparation may be responsible for some of the alterations observed; posttranslational modifications (PTM) and fragmentation influence the binding of the proteins (albumin) to the stationary phase of the depletion column. Nevertheless, in spite of many factors potentially influencing the results, there still may be a relation with MDS itself, since alterations in both ITIH4 and albumin fragments were observed to be related to ovarian carcinoma [20].

A2HSG is a protein of many functions, which have been summarized in the previous study of RA-RARS [9]. Regarding possible functions related to MDS, A2HSG is involved in bone marrow development, inflammation, and cytokine regulations [21–23]. Although A2HSG was only coidentified in two spots in this study, it was chosen for absolute quantification using ELISA, because the 2-DE based results were in agreement with those previously observed in the RA-RARS study, which proved an A2HSG plasma level decrease. In this study, an A2HSG plasma level decrease was observed in RAEB-2 (with statistical significance) and corresponded to an approximately 20% decrease in RAEB-2 when compared to the control group. This quantification result is in accordance with that observed in the RA-RARS group. Moreover, as the plasma level decrease did not correspond to the change observed in spot volumes (in both RA-RARS and RAEB-2 studies), it is likely that there were PTMs influencing the 2-DE results. In a study by Petrik et al. [24], A2HSG was found to reflect the degree of malignancy in different tumor types; therefore, we would expect the lowest plasma level in this RAEB-2 study. Indeed, the average plasma level was estimated to be 438 ± 55 *μ*g/mL for RAEB-2 samples and 471 ± 38 *μ*g/mL for RA-RARS samples. However, it should be noted that in both subgroups the patient cohorts were small, and more importantly there were no spots with A2HSG identification found in the RAEB-1 study at all [11]. Nevertheless, this is the first time it was described that the plasma level of A2HSG is decreased in RAEB-2 patients and, furthermore, that this decrease was further confirmed in another MDS subgroup, RA-RARS.

LRAG is supposed to be a marker of granulocytic differentiation and a protein involved in neutrophil differentiation with upregulated expression [25]. Increased serum concentration of LRAG was found in ulcerative colitis [26], Crohn's disease [27], and nonsmall cell lung cancer [28]; elevated LRAG concentration was observed in the urine of children with acute appendicitis [29]. Recently, Wang et al. [30] revealed that LRAG promotes angiogenesis in mice. Detailed physiological functions of LRAG remain unclear; its properties and possible involvement in pathophysiology have been summarized previously [11]. In this study, LRAG was identified in two spots with a 1.8-fold increase in RAEB-2 patients. From the previous studies, LRAG was identified only in the RAEB-1 study with a 1.5- and 1.7-fold increase in the patient group. According to 2-DE results it seems that the increase in the LRAG plasma level or modification may be related to advanced MDS. In the previous RAEB-1 study, relative quantification of peptides was performed, which revealed the presence of PTM influencing the results. The same peptides were used for relative quantification in this RAEB-2 study. The results however did not correspond to those in RAEB-1 study; both of the quantified peptides showed approximately a 2-fold increase in the patient group. They were thus in agreement with the 2-DE results (1.8-fold increase), indicating LRAG plasma level change. In order to confirm a possible LRAG plasma level change, western blot analysis was performed (Figure 3); the increase in the LRAG plasma level was proven. However, it corresponded to an approximately 30% increase in RAEB-2 and not to the twofold difference. It is known that chemiluminescence detection is a semiquantitative method, and this limitation should be taken into account. Nevertheless, the 30% increase observed by western blot seems to be far lower from the 2-fold increase as estimated by 2-DE and relative quantification. In general, it is proven that there is a change (increase) in the LRAG plasma level in patients with RAEB-2; it is likely that this increase is related to advanced MDS, including both RAEB-1 and RAEB-2 subgroups. Nevertheless, this level of change is not likely to be specific for MDS itself but for related (patho)physiological processes as elevated LRAG serum levels were found in other diseases as we referred above. Further, it is obvious that the PTM influencing the results and presented in RAEB-1 is not present or is dramatically changed in RAEB-2. Thus, it seems that the LRAG plasma level change could be a nonspecific marker of advanced MDS (of processes related to advanced MDS), with a potential to differentiate between the RAEB-1 and RAEB-2 subgroups (according to its PTM); therefore, LRAG modification(s) could be a new biomarker candidate of advanced MDS. However, the potential of differentiating between the two groups would require PTM(s) identification and profiling. Further validation of LRAG as a biomarker of MDS on a large patient cohort will be necessary.

In this study, new potential protein markers for MDS were revealed. Although there are validated tools used for MDS clinical diagnostics and monitoring, they are usually based on cytogenetics or cell morphology that can be time consuming or expensive. In contrast, using protein markers could represent a fast, simple, and low-cost solution. These advantages could be further exploited with the application of surface plasmon resonance technology (SPR). SPR biosensors are capable of detecting multiple biomarkers at once and can be of high sensitivity overcoming traditional ELISA while significantly decreasing the cost and time required for the assay [31–33]. The possibility of multiplexing could be favourably utilized for biomarkers like the one proposed in this study (LRAG) that changes in both the plasma level and its modifications; many protein PTMs could be determined during a single analysis of a sample [34]. Moreover, a minute amount of plasma or even whole blood can be utilized for the assay. These aspects are important especially (but not limited) to oncohematological diseases [35]; blood collection is incomparably easier and more comfortable than bone marrow aspiration.

## 5. Conclusions

In conclusion, in this work new potential myelodysplastic syndrome protein markers were revealed. This study represents a report on the plasma proteome changes in RAEB-2, a high-risk subgroup of MDS and thus completes the picture of plasma protein alterations in different MDS subgroups from low to high risk. Changes in the fragmentation of ITIH4 were observed in the same way as in each of the previously studied MDS subgroups. This is the first study describing that the plasma level of A2HSG, a protein involved in bone marrow development and previously proposed as a MDS biomarker candidate, was significantly decreased in the RAEB-2 subgroup when compared to the control group. Further, an increased expression in RAEB-2 was observed for the first time for LRAG, a protein involved in granulocytic and neutrophil differentiation, and angiogenesis. As the LRAG increased expression was also observed in our previous study of RAEB-1, but not in low-risk MDS subgroups, and it was shown that LRAG modifications differ between the groups, we propose LRAG and its modification(s) as a new biomarker candidate of advanced MDS.

## Figures and Tables

**Table 1:** List of spots that differed significantly when RAEB-2 patients were compared with healthy controls.

Spot	*P*	Fold	Protein	Peptides	AC	SC (%)
1	0.0002	−3.8	Serum albumin	20	P02768	56
			Fibrinogen alpha chain	2	P02671	3
			Hemopexin	2	P02790	20
2	0.008	−3.3	Serum albumin	5	P02768	18
3	0.001	−2.8	Serum albumin	18	P02768	46
			Hemopexin	3	P02790	23
4	0.004	−2.7	Plasma protease C1 inhibitor	5	P05155	15
5	0.0001	−2.4	Alpha-2-HS glycoprotein	2	P02765	17
			Kininogen-1	3	P01042	7
			Corticosteroid-binding globulin	2	P08185	14
6	0.003	−2.4	Serum albumin	5	P02768	13
7	0.007	−2.4	Serum albumin	12	P02768	31
8	0.004	−2.2	Serum albumin	22	P02768	49
9	0.0005	−2.2	Serum albumin	24	P02768	59
10	0.024	2.1	Apolipoprotein A-I	2	P02647	8
			C-reactive protein	4	P02741	20
11	0.008	−2.1	Serum albumin	15	P02768	43
			Protein Z-dependent protease inhibitor	2	Q9UK55	5
12	0.016	2.1	Unidentified			
13	0.017	1.9	Apolipoprotein A-IV	3	P06727	12
			Complement factor I	2	P05156	8
14	0.002	−1.9	Inter-alpha-trypsin inhibitor heavy chain H4	5	Q14624	11
15	0.02	1.9	Serum albumin	6	P02768	16
			Prothrombin	4	P00734	17
			Histidine-rich glycoprotein	2	P04196	4
16	0.001	−1.9	Alpha-2-HS glycoprotein	2	P02765	15
			Kininogen-1	2	P01042	4
17	0.011	−1.9	Serum albumin	11	P02768	26
			Hemopexin	2	P02790	16
18	0.022	−1.9	Serum albumin	7	P02768	13
19	0.0003	1.8	Leucine-rich alpha-2-glycoprotein	7	P02750	30
			Alpha-1-antichymotrypsin	2	P01011	8
20	0.003	1.8	Leucine-rich alpha-2-glycoprotein	5	P02750	28
21	0.039	−1.7	Unidentified			
22	0.002	−1.7	Serum albumin	3	P02768	12
23	0.029	1.7	Haptoglobin	4	P00738	18
24	0.004	1.7	Inter-alpha-trypsin inhibitor heavy chain H4	13	Q14624	20
			Alpha-1-antitrypsin	7	P01009	37
			Angiotensinogen	2	P01019	9
			Prothrombin	2	P00734	14
25	0.006	−1.6	Serum albumin	5	P02768	14
26	0.01	−1.6	Unidentified			
27	0.027	−1.6	Inter-alpha-trypsin inhibitor heavy chain H4	5	Q14624	6
28	0.011	−1.6	Serum albumin	5	P02768	11
			Coagulation factor XII	3	P00748	12
29	0.0008	1.6	Ficolin-2	1	Q15485	4
30	0.02	−1.5	Serum albumin	4	P02768	13
			Apolipoprotein A-IV	2	P06727	7
31	0.0006	−1.5	Clusterin	3	P10909	12
32	0.001	−1.5	Unidentified			
33	0.004	−1.5	Clusterin	2	P10909	7
34	0.016	−1.5	Beta-2-glycoprotein 1	3	P02749	21
35	0.002	−1.5	Prothrombin	7	P00734	26
			Inter-alpha-trypsin inhibitor heavy chain H4	2	Q14624	6
			Alpha-1B-glycoprotein	2	P04217	9
36	0.009	−1.4	Tetranectin	4	P05452	37
37	0.002	−1.4	Tetranectin	2	P05452	16
38	0.0002	1.4	Alpha-1-antichymotrypsin	12	P01011	47
			Corticosteroid-binding globulin	3	P08185	11
39	0.014	−1.4	Alpha-1-antichymotrypsin	3	P01011	11
			Inter-alpha-trypsin inhibitor heavy chain H4	3	Q14624	6
			Clusterin	4	P10909	14
40	0.003	−1.4	Clusterin	4	P10909	14
41	0.017	1.4	Alpha-1-antichymotrypsin	2	P01011	8
			Zinc-alpha-2 glycoprotein	2	P25311	9
42	0.03	−1.3	Inter-alpha-trypsin inhibitor heavy chain H4	4	Q14624	8
43	0.002	−1.3	Clusterin	4	P10909	15
44	0.027	1.3	Inter-alpha-trypsin inhibitor heavy chain H4	13	Q14624	19
			Alpha-1-antitrypsin	3	P01009	18
			Complement component C9	2	P02748	6
45	0.012	−1.3	Clusterin	3	P10909	17
			Inter-alpha-trypsin inhibitor heavy chain H4	2	Q14624	3
46	0.037	−1.3	Clusterin	4	P10909	17
			Inter-alpha-trypsin inhibitor heavy chain H4	3	Q14624	4
47	0.045	1.2	Protein AMBP	2	P02760	12

*P*: ANOVA *P* value; fold: fold difference (multiplication) when comparing RAEB-2 patients and healthy controls (+fold when the normalized volumes increased in RAEB-2); protein: protein identification; peptides: number of unique peptides fulfilling a minimal Mascot score for identity; AC: accession number (SWISS-PROT); SC: protein sequence coverage.
